# Frontotemporal Dementia, Manifested as Schizophrenia, with Decreased Heterochromatin on Chromosome 1

**DOI:** 10.1155/2012/937518

**Published:** 2012-10-02

**Authors:** Philippos Gourzis, Maria Skokou, Panagiotis Polychronopoulos, Evanthia Soubasi, Irene-Eva Triantaphyllidou, Christos Aravidis, Antonia-Ioanna Sarela, Zoe Kosmaidou

**Affiliations:** ^1^Department of Psychiatry, School of Medicine and University Hospital of Patras, University of Patras, 26504 Rio, Patras, Greece; ^2^Department of Neurology, School of Medicine and University Hospital of Patras, University of Patras, 26504 Rio, Patras, Greece; ^3^Department of Chemistry, Laboratory of Biochemistry, University of Patras, 26504 Rio, Patras, Greece; ^4^Department of Genetics, “Alexandra” Hospital, 10671 Athens, Greece

## Abstract

*Introduction*. Frontotemporal dementia is a disorder of complex etiology, with genetic components contributing to the disease. The aim of this report is to describe a young patient suffering from frontotemporal dementia, misdiagnosed as schizophrenia, related to a genetic defect on chromosome 1. *Case Presentation*. A 29-year-old female patient, previously diagnosed as having schizophrenia, was hospitalized with severe behavioural disturbances. She demonstrated severe sexual disinhibition, hyperphagia, lack of motivation, apathy, psychotic symptoms, suicidal thoughts, and cognitive deterioration. Focal atrophy of frontal and anterior temporal structures bilaterally was found on brain MRI, as well as bifrontal hypo perfusion of the brain on SPECT scan. The diagnosis of frontotemporal dementia was made clinically, according to Lund and Manchester groups and Neary diagnostic criteria. Chromosomal analysis was conducted and revealed decrease in length of heterochromatin on the long arm of chromosome 1 (46, XX, 1qh-). Parental karyotypes were normal. *Discussion*. Frontotemporal dementia, and particularly early-onset cases, can be often misdiagnosed as schizophrenia, with negative impact on case management. Genetic testing could be an aid to the correct diagnosis, which is crucial for optimal patient care.

## 1. Introduction

Frontotemporal dementia (FTD) is a mainly presenile degenerative disorder marked by progressive behavioural changes and cognitive impairment, characterized by progressive atrophy of both frontal and anterior temporal lobes [1]. The disease has now been proved as the second most common type of presenile dementia, with a correlation ratio of about one case of FTD to four of Alzheimer's disease (AD) [2]. An autosomal dominant pattern of inheritance is found in 10–27% of all FTD patients [3], with several genetic defects identified in some, but not all, hereditary cases of FTD [4].

FTD includes a heterogeneous group of sporadic and familial neuropsychiatric disorders [5]. The age of onset is most often between 45–65 years (range 21–85) and the mean duration of illness is 8 years, ranging from 2 to 20 years [2]. Since it mainly affects people in midlife, presenting with behavioural disorder and personality change, it is of special importance to psychiatry [6]. According to presenting symptoms, it can be mistaken for depression, mania [1], or schizophrenia, particularly in younger onset cases [7].

Differentiation between FTD, primary psychiatric disorders, AD, and other dementias is crucial for case management and determining prognosis. Identification of factors relating to etiology, age of onset, and clinical presentation of FTD could help expand our understanding of the pathogenesis of the disorder, and probably enhance accurate clinical diagnosis [8, 9].

## 2. Case Presentation

A 29-year-old woman with a five-year history of schizophrenia was admitted in the psychiatric clinic of our institution, due to suicidal ideation, atypical symptoms, and unresponsiveness to treatment. She was until then treated with antipsychotic drugs, namely risperidone, olanzapine, and trifluoperazine, as well as antidepressants and benzodiazepines, without any improvement.

The patient's history had begun five years ago and consisted of gradual personality and behavioural changes. She initially presented sexual disinhibition disturbing her closest friend, of the same sex, with several calls. After that she was stopping strangers passing by in order to inform them about her sexual preferences and asking their opinion about them. The same period she began to wander far away from home, spending many hours in coffee bars—doing nothing. She presented anxiety, depressed mood, and mood lability with frequent transitions between laughter and tears and somatic complaints of pain in various parts of her body. She gradually began to neglect her personal hygiene and demonstrated compulsive behaviour, wetting her face many times throughout the day, explaining that her friend would see her and think that she was crying for her. Delusional ideas of bizarre content were also developed, such as that there were many small lights on her body. On accidentally being touched by others she became extremely worried, as she thought that this would cause removal of some lights, and when all lights would be removed, she would die. One year before admission, the delusional ideas remitted; however she demonstrated suicidal thoughts, hyperphagia, and severe sexual disinhibition. She started asking from her familiar persons, including her mother, father, and grandmother to have sex with her. Because of her grossly disturbed behavior and suicidal ideation she was hospitalized.

On admission she was agitated and fearful, frequently asking for the doctor's reassurance that she was not going to die. Her verbal output decreased. Her speech was not spontaneous, and she only gave brief, monosyllabic answers. She tended to give repetitive “don't know” replies; she was giggling inappropriately and exhibited mannerisms such as humming and foot tapping. She kept asking whether she was going to die and had no insight. 

She had no past medical or psychiatric history. Also, she had no history of alcohol or illegal drug abuse or head injury. She had been delivered normally and developmental milestones were also normal. She had a normal school life. After school, she had few friends and used to work occasionally as a housekeeper, until she became ill. There was no family history for dementia or psychosis.

A full range of examinations, including blood, metabolic, hormonal, and cerebrospinal fluid tests, yielded normal results. Serum ceruloplasmin levels were normal. Physical examination was entirely normal. Ophthalmologic assessment including funduscopy did not reveal any abnormalities. EEG was within normal limits. Brain MRI showed focal atrophy of frontal and anterior temporal structures bilaterally. SPECT scan demonstrated bifrontal hypo perfusion of the brain. Assessment with the Wechsler Adult Intelligence Scale (WAIS) yielded a full-scale score of intelligence quotient (IQ) of 64. In the Wisconsin Card Sorting Test she achieved very low performance. Neurological examination was otherwise normal. A clinical diagnosis of frontotemporal dementia was made according to Lund and Manchester groups [10] and Neary diagnostic criteria [11].

**Figure 1:**
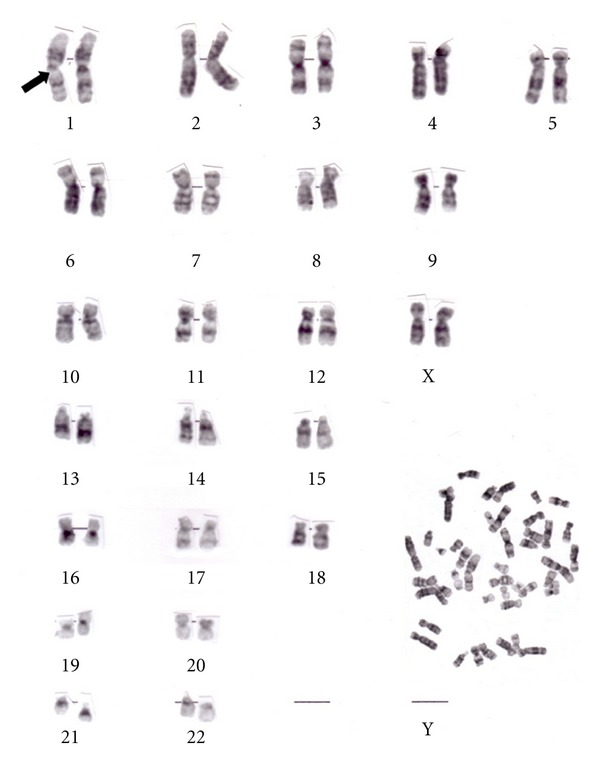
Decrease of the heterochromatin on the long arm of chromosome 1 (46, XX, 1qh-).

Analysis of the patient's karyotype revealed a decrease in length of the heterochromatin on the long arm of chromosome 1 (46, XX, 1qh-) (Figure 1). The karyotypes of her parents were normal. The patient was followed up for at least two years after discharge, showing further slow deterioration.

## 3. Discussion

Because of young age of onset, presence of delusions, and what was thought to be negative symptoms of the illness, the patient was previously misdiagnosed as having schizophrenia. However, the progressive personality change and behavioural disturbance, with marked disinhibition, apathy, personal neglect, and features of Kluver-Bucy syndrome—hyperphagia, hypersexuality—along with cognitive decline, language dysfunction, and neuroimaging findings warranted a clinical diagnosis of FTD, according to Lund and Manchester groups [10] and Neary diagnostic criteria [11]. There was a five-year delay of correct diagnosis, after the onset of symptomatology. Furthermore, to our knowledge this is the first case of early onset FTD that is related to a de novo decrease in length of the heterochromatin on the long arm of chromosome 1 (46, XX.1qh-) (Figure 1).

Frontotemporal lobar degeneration (FTLD) is characterized by progressive, circumscribed atrophy of frontal and temporal lobe cortices [1], which was demonstrated by MRI imaging in the present case, but other structures such as the basal ganglia and the cortico-striato-pallido-thalamo-cortical loops seem to be involved as well [12]. The selective frontal and/or temporal reduction in blood flow or metabolism can be detected by SPECT or PET scan [1], as has been also found in our patient. The pathological changes result in the clinical syndrome of FTD, with gradual, deteriorating changes in behavior, personality, and/or language, whereas memory is relatively preserved in the early stages of the illness. The age of onset is typically between 45–65 years; however pathologically proven cases have been recorded in individuals as young as 21 years old [2]. It encompasses three distinct clinical syndromes: behavioural variant FTD (bvFTD), semantic dementia, and progressive nonfluent aphasia, while there is also overlap with motor neuron disease, and the parkinsonian syndromes, corticobasal degeneration (CBD), and progressive supranuclear palsy (PSP) [3]. FTLD can be divided into two major subtypes, with tau positive inclusions (FTLD-tau), and with ubiquitin positive, transactive response DNA binding protein- (TDP-43) positive, but tau-negative inclusions (FTLD-TDP) [3, 13].

The clinical and pathological variance of FTLD may reflect different phenotypic expression of particular genetic changes. Mutations in the gene for microtubule-associated protein tau (*MAPT*) on chromosome 17 is responsible for 10–20% of familial cases and results in tau-positive inclusions pathology, while mutations in the progranulin (*PGRN*) gene, also on chromosome 17, is the cause for many other cases, resulting in frontotemporal dementia with the presence of ubiquitin positive inclusions containing TDP-43, accounting for 5–10% of cases [3, 14–17]. Less common mutations are found in the gene for valosin-containing protein (*VCP*) on 9p21-12 [18], in the gene coding for TDP-43 [19], in the gene for charged multivesicular binding protein 2B (*CHMP2B*) [20], and in the fused-in-sarcoma (*FUS*) gene on chromosome 16 [21], but there still remain cases with as yet unidentified genetic defects. Regarding age of onset of FTD, *FUS* gene mutations have been associated with age of onset ≤40 years of age [21]; on the other hand, early onset cases of alzheimer disease (AD) have been reported to be associated with mutations in the genes encoding for presenilin 1 (*PS1*) on chromosome 14, presenilin 2 (*PS2*) on chromosome 1, and the amyloid *β*-protein precursor (*APP*) on chromosome 21 [13].

FTD in a remarkably young patient has to be diagnosed with caution. It is necessary to rule out other causes of early adulthood dementia and psychosis as Wilson's disease, leukodystrophies, variant Creutzfeldt-Jacob disease, storage or mitochondrial diseases. The detailed-physical, neurological, laboratory, and neuroimaging diagnostic tests of our patient on admission and after two years of followup did not reveal anything of the above.

On the contrary, early onset of FTD may result, in early stages, in misdiagnosing such a patient as having another primary psychiatric disorder, particularly schizophrenia, given that both share common areas of brain damage [22]. FTD patients with psychosis seem to be younger at onset, and experience a longer delay between onset and subsequent presentation. Young persons presenting unusual behaviour, emotional blunting, social withdrawal, declining psychosocial functioning, and executive dysfunction with or without frontotemporal changes on imaging are likely to receive a psychiatric diagnosis of schizophrenia [7]. Furthermore, the existence of a group of patients with “nonprogressive” or “slow” bvFTD, with only slow progress of symptoms and a longer than usual life expectancy [3], raises the possibility that a larger than currently suspected proportion of patients diagnosed as having schizophrenia actually suffer from FTD.

Chromosome 1 is also implicated in early onset of Alzheimer's disease through mutation in presenilin 2 gene (*PS2*) [23], as well as in infantile neuronal ceroid lipofuscinosis, which presents with dementia, among other core symptoms [24], but there is no such finding relating chromosome 1 defect with FTD until presently. Genetic findings could be an aid to the correct diagnosis, since current criteria lack sensitivity in the early stages of the disease [25]. Particularly regarding early onset cases of FTD, chromosomal analysis may be a useful marker for diagnosis, since alterations associated with chromosome 1 are implicated in the pathogenesis of early onset AD as well, and might represent a factor modifying age of onset in some cases of dementia.

## Abbreviation List

AD: Alzheimer disease
APP: Amyloid *β* protein precursor
bvFTD: Behavioural-variant frontotemporal dementia
CBD: Corticobasal degeneration
CHMP2B: Charged multivesicular binding protein 2B
EEG: Electroencephalogram
FTD: Frontotemporal dementia
FTLD: Frontotemporal lobar degeneration
FUS: Fused in sarcoma
IQ: Intelligence quotient
MAPT: Microtubule-associated protein tau
MRI: Magnetic resonance imaging
PGRN: progranulin gene
PSP: Progressive supranuclear palsy
SPECT: Single-photon emission computed tomography
TDP-43: Transactive response deoxyribonucleic acid binding protein-43 Kd
VCP: Valosin-containing protein
WAIS: Wechsler Adult Intelligence Scale.

## References

[1] Richards SS, Sweet RA, Sadock BJ, Sadock VA, Ruiz P (2009). Dementia. *Comprehensive Textbook of Psychiatry*.

[2] Neary D, Snowden J, Mann D (2005). Frontotemporal dementia. *The Lancet Neurology*.

[3] Seelaar H, Rohrer JD, Pijnenburg YAL, Fox NC, Van Swieten JC (2011). Clinical, genetic and pathological heterogeneity of frontotemporal dementia: a review. *Journal of Neurology, Neurosurgery and Psychiatry*.

[4] van Swieten JC, Heutink P (2008). Mutations in progranulin (GRN) within the spectrum of clinical and pathological phenotypes of frontotemporal dementia. *The Lancet Neurology*.

[5] McKhann GM, Albert MS, Grossman M, Miller B, Dickson D, Trojanowski JQ (2001). Clinical and pathological diagnosis of frontotemporal dementia: report of the work group on Frontotemporal Dementia and Pick’s Disease. *Archives of Neurology*.

[6] Lovestone S, David AS, Fleminger S, Kopelman MD, Lovestone S, Mellers JDC (2009). Alzheimer's disease and other dementias (including pseudodementias). *Lishman's Organic Psychiatry: a Textbook of Neuropsychiatry*.

[7] Velakoulis D, Walterfang M, Mocellin R, Pantelis C, McLean C (2009). Frontotemporal dementia presenting as schizophrenia-like psychosis in young people: clinicopathological series and review of cases. *British Journal of Psychiatry*.

[8] Varma AR, Snowden JS, Lloyd JJ, Talbot PR, Mann DMA, Neary D (1999). Evaluation of the NINCDS-ADRDA criteria in the differentiation of Alzheimer’s disease and frontotemporal dementia. *Journal of Neurology Neurosurgery and Psychiatry*.

[9] Van Deerlin VM, Gill LH, Farmer JM, Trojanowski JQ, Lee VMY (2003). Familial frontotemporal dementia: from gene discovery to clinical molecular diagnostics. *Clinical Chemistry*.

[10] Brun A, Englund B, Gustafson L (1994). Clinical and neuropathological criteria for frontotemporal dementia. *Journal of Neurology Neurosurgery and Psychiatry*.

[11] Neary D, Snowden JS, Gustafson L (1998). Frontotemporal lobar degeneration: a consensus on clinical diagnostic criteria. *Neurology*.

[12] Looi JC, Walterfang M, Velacoulis D, MacFarlane MD, Svensson LA, Wahlund LO (2012). Frontotemporal dementia as a frontostriatal disorder: neostriatal morphology as a biomarker and structural basis for an endophenotype. *Australian & New Zealand Journal of Psychiatry*.

[13] Blacker D, Tanzi RE (1998). The genetics of Alzheimer disease: current status and future prospects. *Archives of Neurology*.

[14] Poorkaj P, Grossman M, Steinbart E (2001). Frequency of tau gene mutations in familial and sporadic cases of non-Alzheimer dementia. *Archives of Neurology*.

[15] Rademakers R, Cruts M, Van Broeckhoven C (2004). The role of tau (MAPT) in frontotemporal dementia and related tauopathies. *Human Mutation*.

[16] Baker M, Mackenzie IR, Pickering-Brown SM (2006). Mutations in progranulin cause tau-negative frontotemporal dementia linked to chromosome 17. *Nature*.

[17] Cruts M, Gijselinck I, Van Der Zee J (2006). Null mutations in progranulin cause ubiquitin-positive frontotemporal dementia linked to chromosome 17q21. *Nature*.

[18] Watts GDJ, Wymer J, Kovach MJ (2004). Inclusion body myopathy associated with Paget disease of bone and frontotemporal dementia is caused by mutant valosin-containing protein. *Nature Genetics*.

[19] Borroni B, Bonvicini C, Alberici A (2009). Mutation within TARDBP leads to frontotemporal dementia without motor neuron disease. *Human Mutation*.

[20] Gydesen S, Brown JM, Brun A (2002). Chromosome 3 linked frontotemporal dementia (FTD-3). *Neurology*.

[21] Van Langenhove T, Van Der Zee J, Sleegers K (2010). Genetic contribution of FUS to frontotemporal lobar degeneration. *Neurology*.

[22] Stone J, Griffiths TD, Rastogi S, Perry RH, Cleland PG (2003). Non-picks frontotemporal dementia imitating schizophrenia in a 22-year-old man. *Journal of Neurology*.

[23] Tanzi RE, Kovacs DM, Kim TW, Moir RD, Guenette SY, Wasco W (1996). The gene defects responsible for familial Alzheimer’s disease. *Neurobiology of Disease*.

[24] Goebel HH (1995). The neuronal ceroid-lipofuscinoses. *Journal of Child Neurology*.

[25] Mendez MF, Shapira JS, McMurtray A, Licht E, Miller BL (2007). Accuracy of the clinical evaluation for frontotemporal dementia. *Archives of Neurology*.

